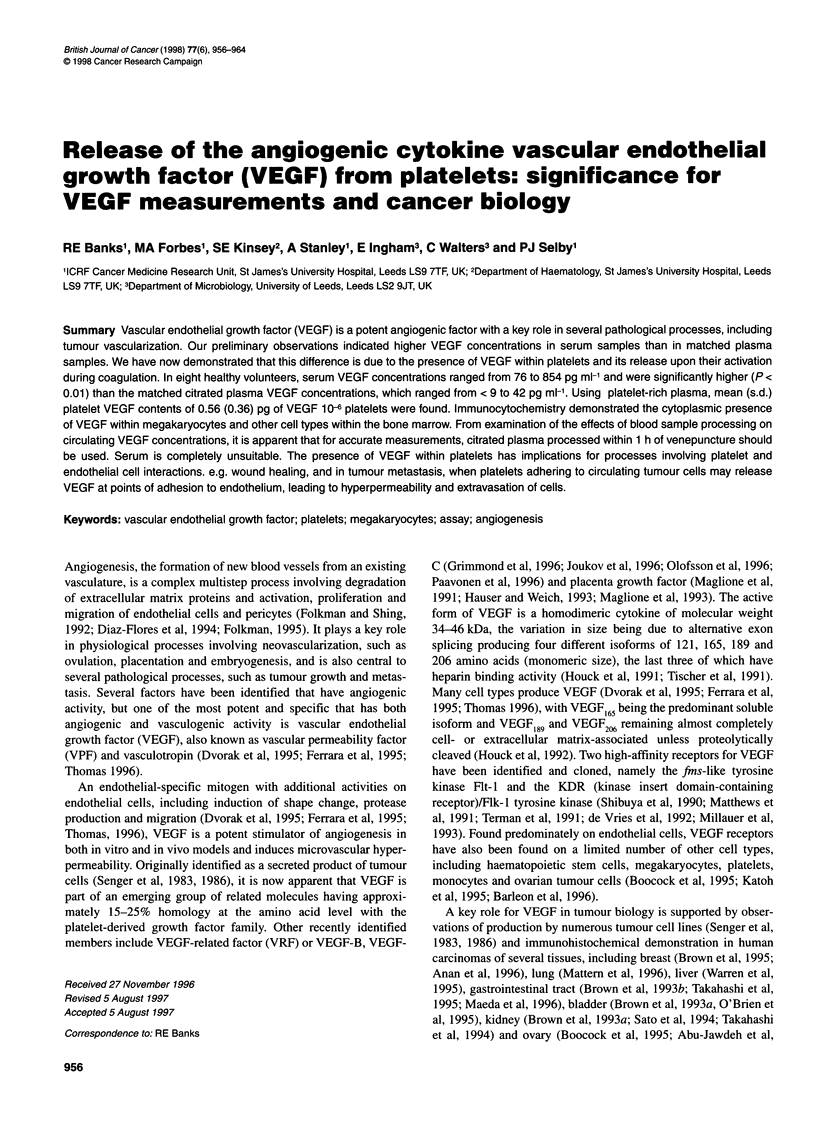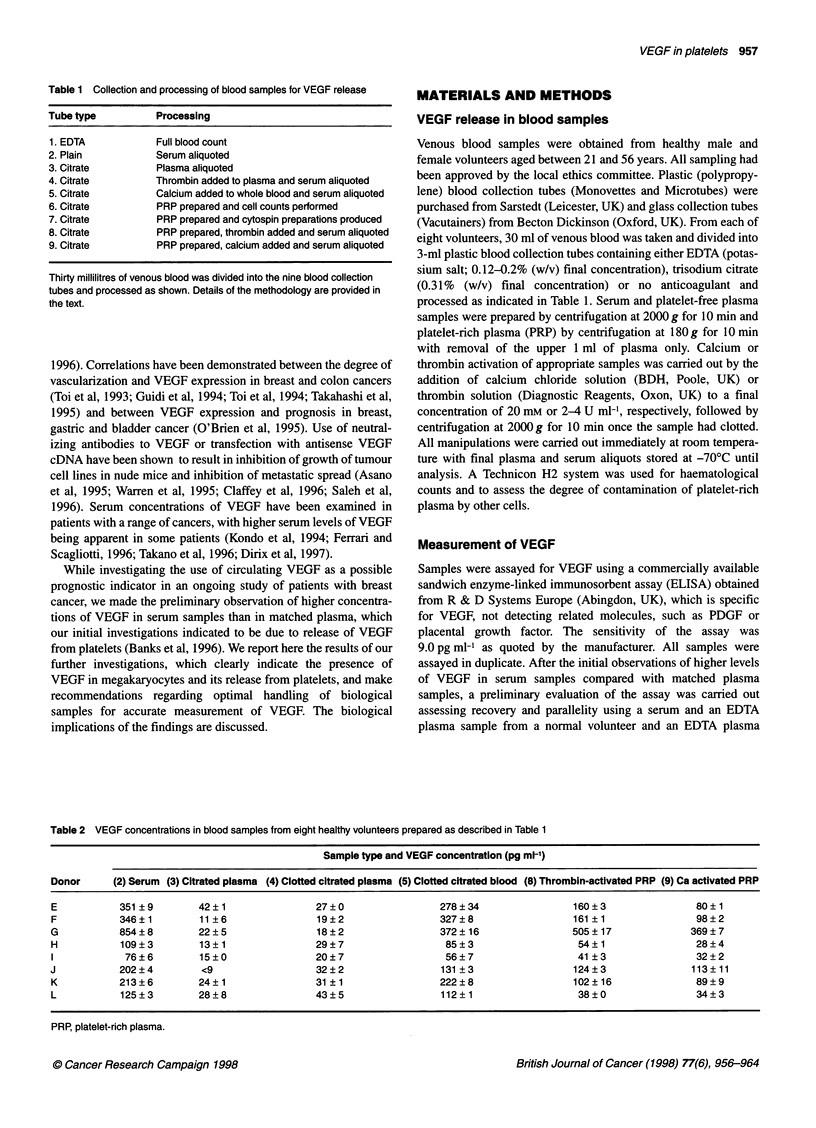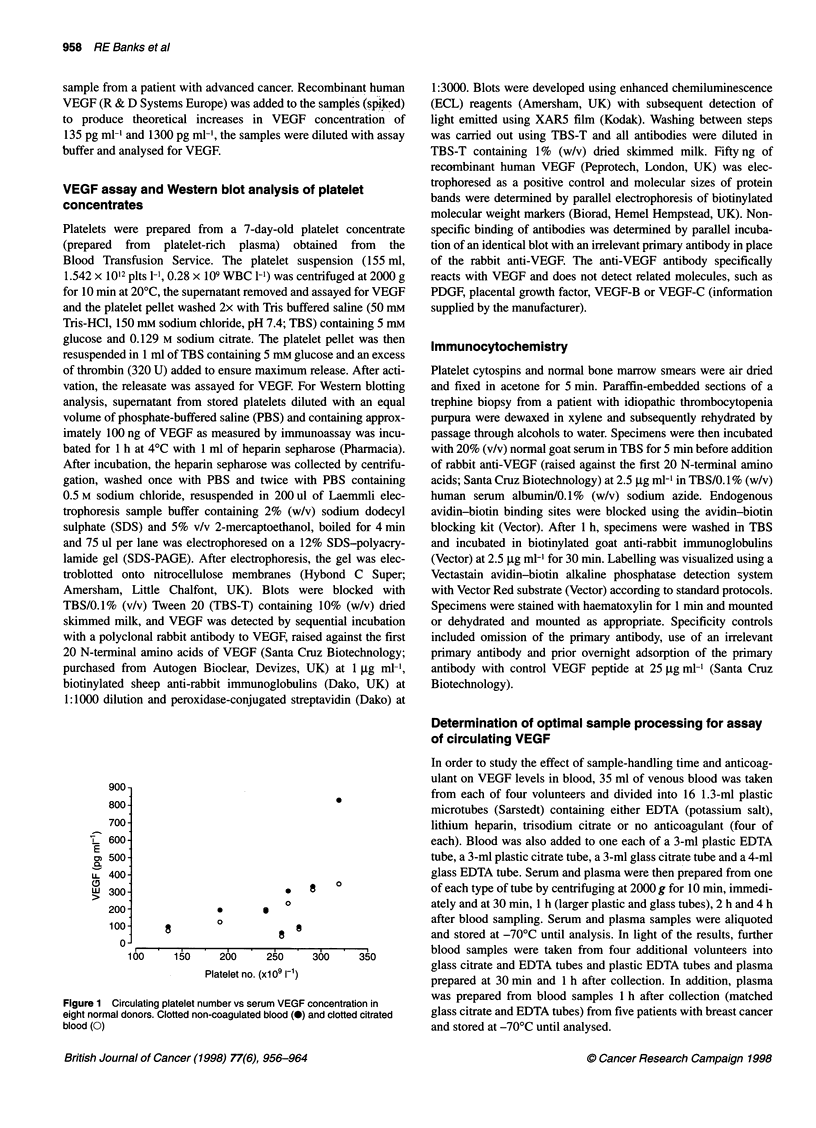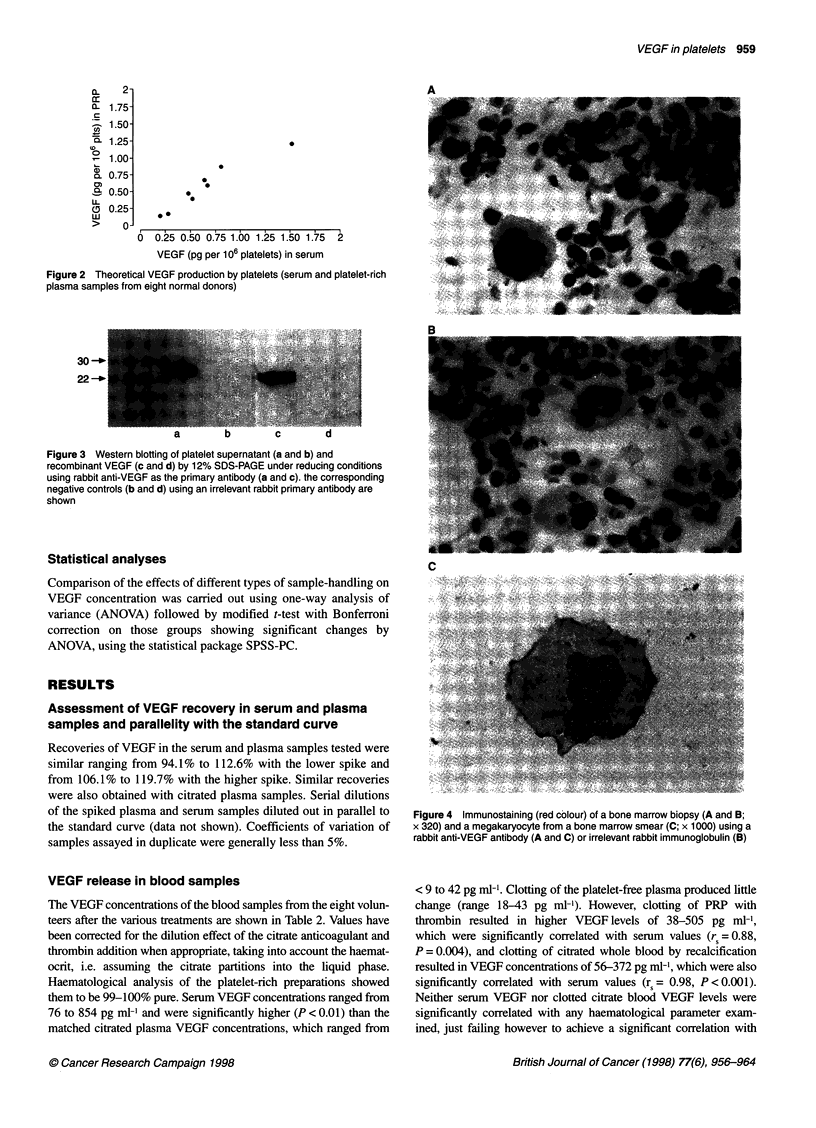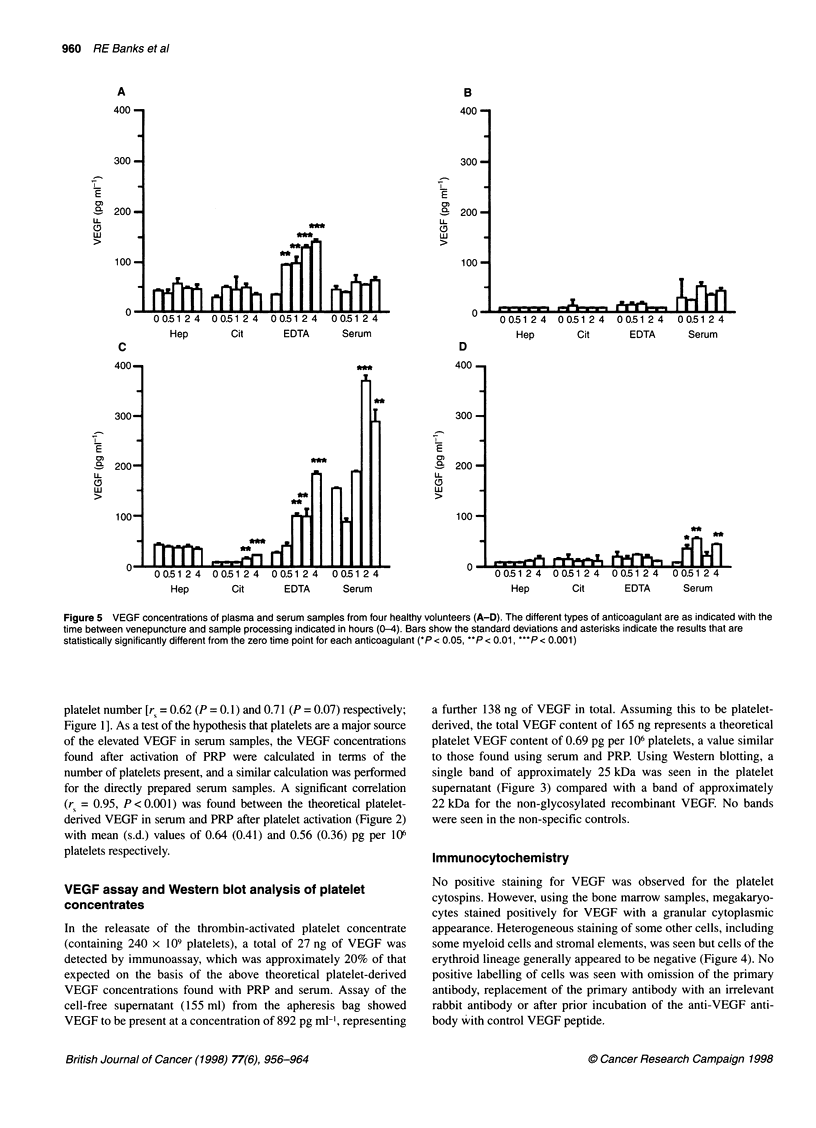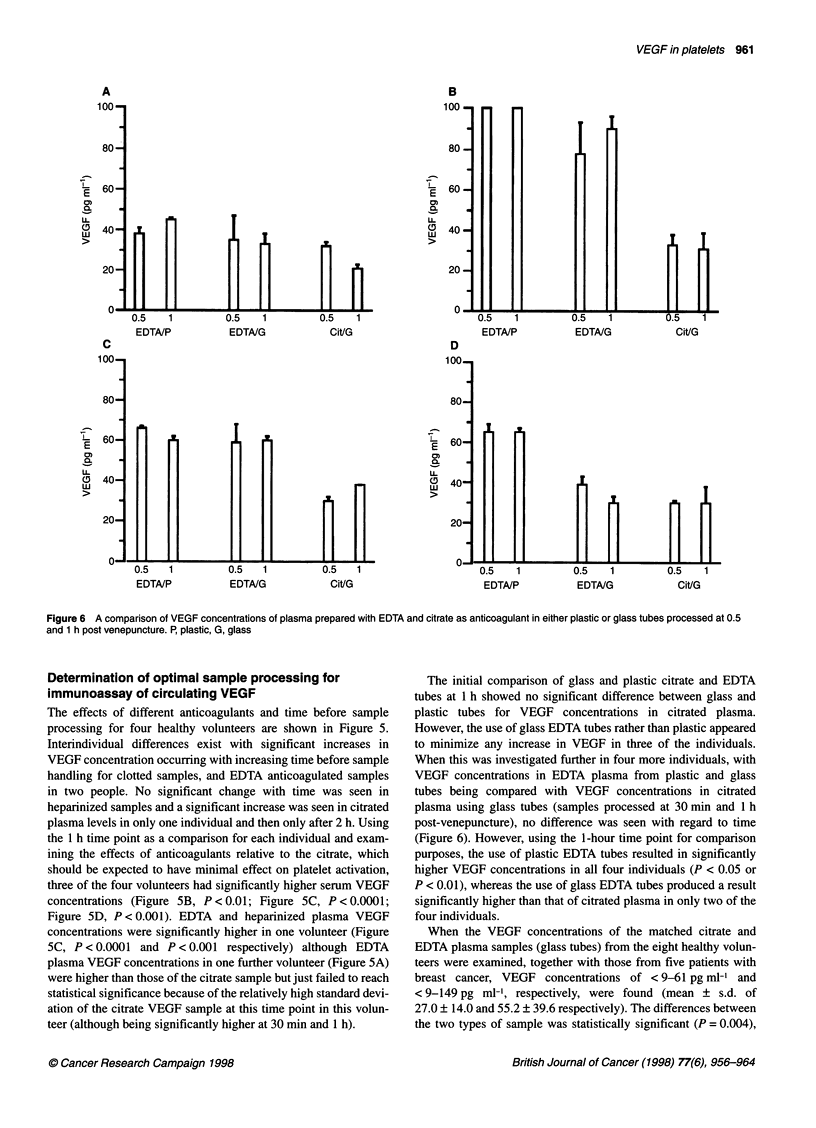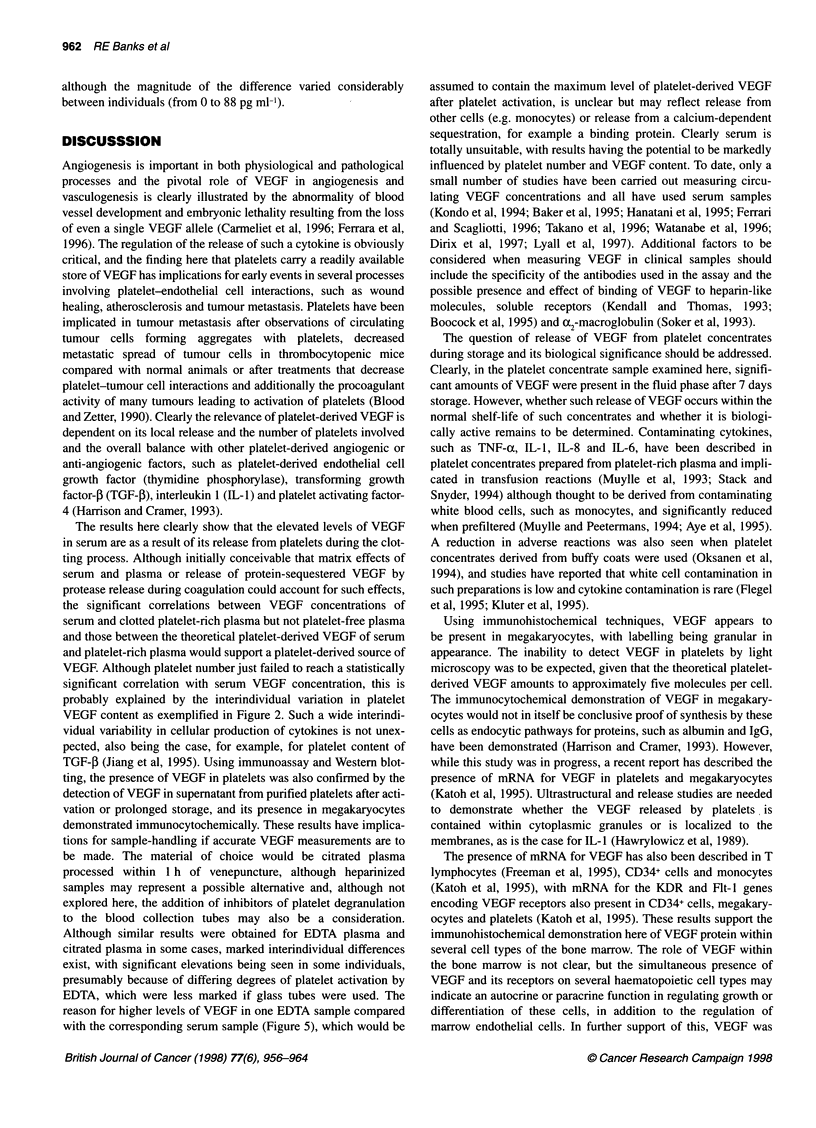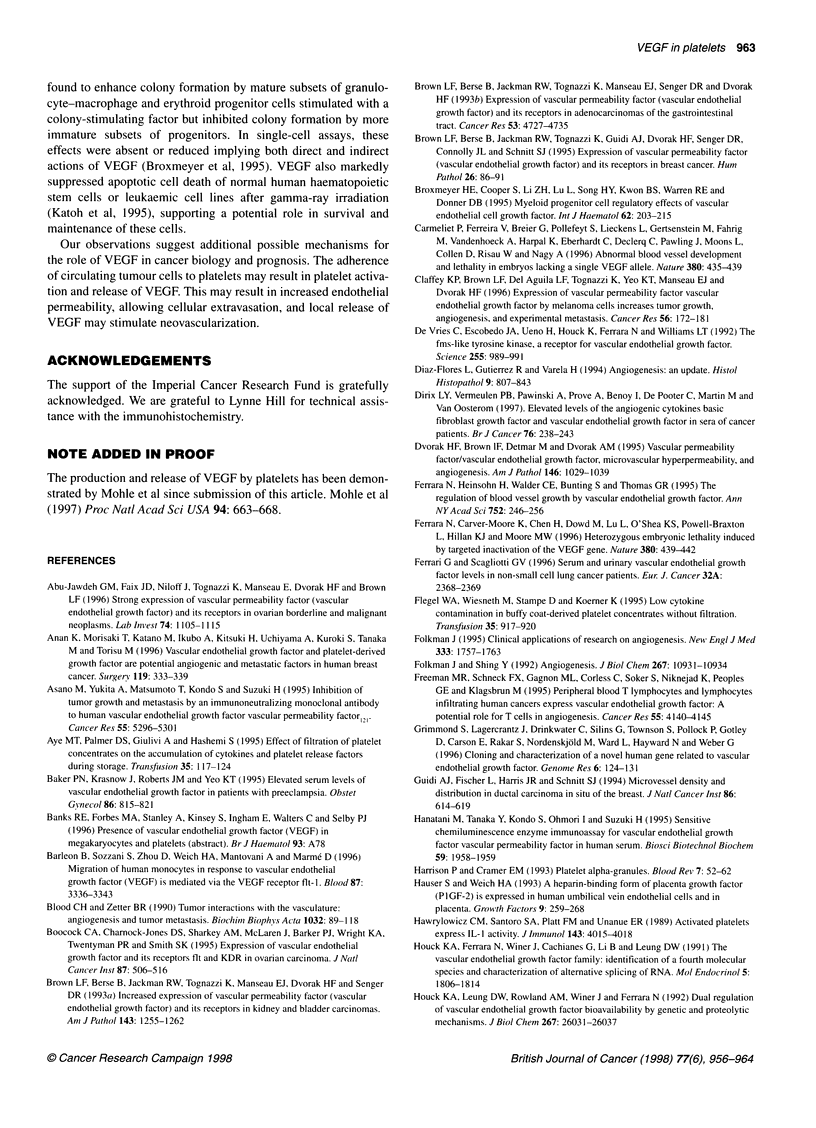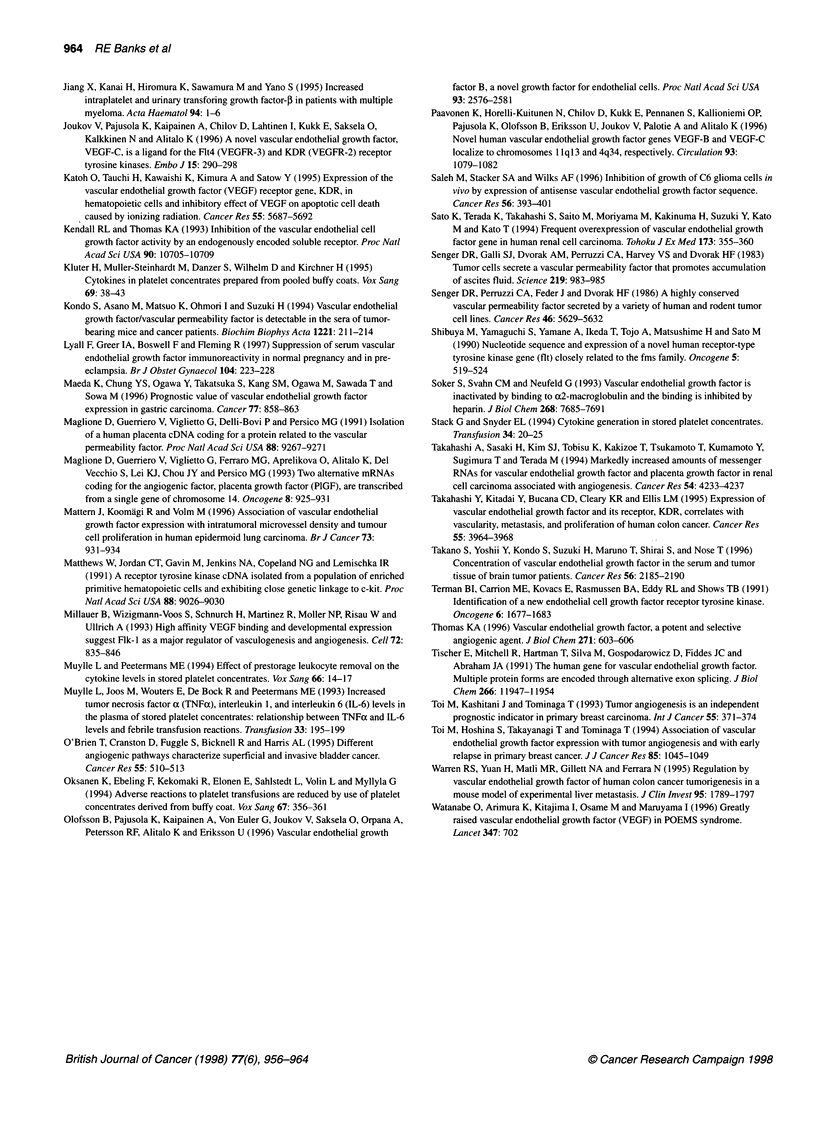# Release of the angiogenic cytokine vascular endothelial growth factor (VEGF) from platelets: significance for VEGF measurements and cancer biology.

**DOI:** 10.1038/bjc.1998.158

**Published:** 1998-03

**Authors:** R. E. Banks, M. A. Forbes, S. E. Kinsey, A. Stanley, E. Ingham, C. Walters, P. J. Selby

**Affiliations:** ICRF Cancer Medicine Research Unit, St James's University Hospital, Leeds UK.

## Abstract

**Images:**


British Journal of Cancer (1998) 77(6), 956–964
© 1998 Cancer Research Campaign

### Release of the angiogenic cytokine vascular endothelial growth factor (VEGF) from platelets: significance for VEGF measurements and cancer biology

**RE Banks[1], MA Forbes[1], SE Kinsey[2], A Stanley[1], E Ingham[3], C Walters[3] and PJ Selby[1]**

[1]ICRF Cancer Medicine Research Unit, St James's University Hospital, Leeds LS9 7TF, UK; [2]Department of Haematology, St James's University Hospital, Leeds LS9 7TF, UK; [3]Department of Microbiology, University of Leeds, Leeds LS2 9JT, UK

**Summary** Vascular endothelial growth factor (VEGF) is a potent angiogenic factor with a key role in several pathological processes, including tumour vascularization. Our preliminary observations indicated higher VEGF concentrations in serum samples than in matched plasma samples. We have now demonstrated that this difference is due to the presence of VEGF within platelets and its release upon their activation during coagulation. In eight healthy volunteers, serum VEGF concentrations ranged from 76 to 854 pg $ml^{-1}$ and were significantly higher ($P < 0.01$) than the matched citrated plasma VEGF concentrations, which ranged from < 9 to 42 pg $ml^{-1}$. Using platelet-rich plasma, mean (s.d.) platelet VEGF contents of 0.56 (0.36) pg of VEGF $10^{-6}$ platelets were found. Immunocytochemistry demonstrated the cytoplasmic presence of VEGF within megakaryocytes and other cell types within the bone marrow. From examination of the effects of blood sample processing on circulating VEGF concentrations, it is apparent that for accurate measurements, citrated plasma processed within 1 h of venepuncture should be used. Serum is completely unsuitable. The presence of VEGF within platelets has implications for processes involving platelet and endothelial cell interactions. e.g. wound healing, and in tumour metastasis, when platelets adhering to circulating tumour cells may release VEGF at points of adhesion to endothelium, leading to hyperpermeability and extravasation of cells.

**Keywords:** vascular endothelial growth factor; platelets; megakaryocytes; assay; angiogenesis

Angiogenesis, the formation of new blood vessels from an existing vasculature, is a complex multistep process involving degradation of extracellular matrix proteins and activation, proliferation and migration of endothelial cells and pericytes (Folkman and Shing, 1992; Diaz-Flores et al, 1994; Folkman, 1995). It plays a key role in physiological processes involving neovascularization, such as ovulation, placentation and embryogenesis, and is also central to several pathological processes, such as tumour growth and metastasis. Several factors have been identified that have angiogenic activity, but one of the most potent and specific that has both angiogenic and vasculogenic activity is vascular endothelial growth factor (VEGF), also known as vascular permeability factor (VPF) and vasculotropin (Dvorak et al, 1995; Ferrara et al, 1995; Thomas 1996).

An endothelial-specific mitogen with additional activities on endothelial cells, including induction of shape change, protease production and migration (Dvorak et al, 1995; Ferrara et al, 1995; Thomas, 1996), VEGF is a potent stimulator of angiogenesis in both in vitro and in vivo models and induces microvascular hyperpermeability. Originally identified as a secreted product of tumour cells (Senger et al, 1983, 1986), it is now apparent that VEGF is part of an emerging group of related molecules having approximately 15–25% homology at the amino acid level with the platelet-derived growth factor family. Other recently identified members include VEGF-related factor (VRF) or VEGF-B, VEGF-C (Grimmond et al, 1996; Joukov et al, 1996; Olofsson et al, 1996; Paavonen et al, 1996) and placenta growth factor (Maglione et al, 1991; Hauser and Weich, 1993; Maglione et al, 1993). The active form of VEGF is a homodimeric cytokine of molecular weight 34–46 kDa, the variation in size being due to alternative exon splicing producing four different isoforms of 121, 165, 189 and 206 amino acids (monomeric size), the last three of which have heparin binding activity (Houck et al, 1991; Tischer et al, 1991). Many cell types produce VEGF (Dvorak et al, 1995; Ferrara et al, 1995; Thomas 1996), with $VEGF_{165}$ being the predominant soluble isoform and $VEGF_{189}$ and $VEGF_{206}$ remaining almost completely cell- or extracellular matrix-associated unless proteolytically cleaved (Houck et al, 1992). Two high-affinity receptors for VEGF have been identified and cloned, namely the *fms*-like tyrosine kinase Flt-1 and the KDR (kinase insert domain-containing receptor)/Flk-1 tyrosine kinase (Shibuya et al, 1990; Matthews et al, 1991; Terman et al, 1991; de Vries et al, 1992; Millauer et al, 1993). Found predominately on endothelial cells, VEGF receptors have also been found on a limited number of other cell types, including haematopoietic stem cells, megakaryocytes, platelets, monocytes and ovarian tumour cells (Boocock et al, 1995; Katoh et al, 1995; Barleon et al, 1996).

A key role for VEGF in tumour biology is supported by observations of production by numerous tumour cell lines (Senger et al, 1983, 1986) and immunohistochemical demonstration in human carcinomas of several tissues, including breast (Brown et al, 1995; Anan et al, 1996), lung (Mattern et al, 1996), liver (Warren et al, 1995), gastrointestinal tract (Brown et al, 1993*b*; Takahashi et al, 1995; Maeda et al, 1996), bladder (Brown et al, 1993*a*, O'Brien et al, 1995), kidney (Brown et al, 1993*a*; Sato et al, 1994; Takahashi et al, 1994) and ovary (Boocock et al, 1995; Abu-Jawdeh et al,

*Received 27 November 1996*
*Revised 5 August 1997*
*Accepted 5 August 1997*

*Correspondence to:* RE Banks

Table 1 Collection and processing of blood samples for VEGF release

| Tube type | Processing |
|---|---|
| 1. EDTA | Full blood count |
| 2. Plain | Serum aliquoted |
| 3. Citrate | Plasma aliquoted |
| 4. Citrate | Thrombin added to plasma and serum aliquoted |
| 5. Citrate | Calcium added to whole blood and serum aliquoted |
| 6. Citrate | PRP prepared and cell counts performed |
| 7. Citrate | PRP prepared and cytospin preparations produced |
| 8. Citrate | PRP prepared, thrombin added and serum aliquoted |
| 9. Citrate | PRP prepared, calcium added and serum aliquoted |

Thirty millilitres of venous blood was divided into the nine blood collection tubes and processed as shown. Details of the methodology are provided in the text.

1996). Correlations have been demonstrated between the degree of vascularization and VEGF expression in breast and colon cancers (Toi et al, 1993; Guidi et al, 1994; Toi et al, 1994; Takahashi et al, 1995) and between VEGF expression and prognosis in breast, gastric and bladder cancer (O'Brien et al, 1995). Use of neutralizing antibodies to VEGF or transfection with antisense VEGF cDNA have been shown to result in inhibition of growth of tumour cell lines in nude mice and inhibition of metastatic spread (Asano et al, 1995; Warren et al, 1995; Claffey et al, 1996; Saleh et al, 1996). Serum concentrations of VEGF have been examined in patients with a range of cancers, with higher serum levels of VEGF being apparent in some patients (Kondo et al, 1994; Ferrari and Scagliotti, 1996; Takano et al, 1996; Dirix et al, 1997).

While investigating the use of circulating VEGF as a possible prognostic indicator in an ongoing study of patients with breast cancer, we made the preliminary observation of higher concentrations of VEGF in serum samples than in matched plasma, which our initial investigations indicated to be due to release of VEGF from platelets (Banks et al, 1996). We report here the results of our further investigations, which clearly indicate the presence of VEGF in megakaryocytes and its release from platelets, and make recommendations regarding optimal handling of biological samples for accurate measurement of VEGF. The biological implications of the findings are discussed.

### MATERIALS AND METHODS

### VEGF release in blood samples

Venous blood samples were obtained from healthy male and female volunteers aged between 21 and 56 years. All sampling had been approved by the local ethics committee. Plastic (polypropylene) blood collection tubes (Monovettes and Microtubes) were purchased from Sarstedt (Leicester, UK) and glass collection tubes (Vacutainers) from Becton Dickinson (Oxford, UK). From each of eight volunteers, 30 ml of venous blood was taken and divided into 3-ml plastic blood collection tubes containing either EDTA (potassium salt; 0.12–0.2% (w/v) final concentration), trisodium citrate (0.31% (w/v) final concentration) or no anticoagulant and processed as indicated in Table 1. Serum and platelet-free plasma samples were prepared by centrifugation at 2000 *g* for 10 min and platelet-rich plasma (PRP) by centrifugation at 180 *g* for 10 min with removal of the upper 1 ml of plasma only. Calcium or thrombin activation of appropriate samples was carried out by the addition of calcium chloride solution (BDH, Poole, UK) or thrombin solution (Diagnostic Reagents, Oxon, UK) to a final concentration of 20 mM or 2–4 U $ml^{-1}$, respectively, followed by centrifugation at 2000 *g* for 10 min once the sample had clotted. All manipulations were carried out immediately at room temperature with final plasma and serum aliquots stored at –70°C until analysis. A Technicon H2 system was used for haematological counts and to assess the degree of contamination of platelet-rich plasma by other cells.

### Measurement of VEGF

Samples were assayed for VEGF using a commercially available sandwich enzyme-linked immunosorbent assay (ELISA) obtained from R & D Systems Europe (Abingdon, UK), which is specific for VEGF, not detecting related molecules, such as PDGF or placental growth factor. The sensitivity of the assay was 9.0 pg $ml^{-1}$ as quoted by the manufacturer. All samples were assayed in duplicate. After the initial observations of higher levels of VEGF in serum samples compared with matched plasma samples, a preliminary evaluation of the assay was carried out assessing recovery and parallelity using a serum and an EDTA plasma sample from a normal volunteer and an EDTA plasma

Table 2 VEGF concentrations in blood samples from eight healthy volunteers prepared as described in Table 1

| | Sample type and VEGF concentration (pg $ml^{-1}$) | | | | | |
|---|---|---|---|---|---|---|
| Donor | (2) Serum | (3) Citrated plasma | (4) Clotted citrated plasma | (5) Clotted citrated blood | (8) Thrombin-activated PRP | (9) Ca activated PRP |
| E | 351 ± 9 | 42 ± 1 | 27 ± 0 | 278 ± 34 | 160 ± 3 | 80 ± 1 |
| F | 346 ± 1 | 11 ± 6 | 19 ± 2 | 327 ± 8 | 161 ± 1 | 98 ± 2 |
| G | 854 ± 8 | 22 ± 5 | 18 ± 2 | 372 ± 16 | 505 ± 17 | 369 ± 7 |
| H | 109 ± 3 | 13 ± 1 | 29 ± 7 | 85 ± 3 | 54 ± 1 | 28 ± 4 |
| I | 76 ± 6 | 15 ± 0 | 20 ± 7 | 56 ± 7 | 41 ± 3 | 32 ± 2 |
| J | 202 ± 4 | <9 | 32 ± 2 | 131 ± 3 | 124 ± 3 | 113 ± 11 |
| K | 213 ± 6 | 24 ± 1 | 31 ± 1 | 222 ± 8 | 102 ± 16 | 89 ± 9 |
| L | 125 ± 3 | 28 ± 8 | 43 ± 5 | 112 ± 1 | 38 ± 0 | 34 ± 3 |

PRP, platelet-rich plasma.

© Cancer Research Campaign 1998

sample from a patient with advanced cancer. Recombinant human VEGF (R & D Systems Europe) was added to the samples (spiked) to produce theoretical increases in VEGF concentration of 135 pg $ml^{-1}$ and 1300 pg $ml^{-1}$, the samples were diluted with assay buffer and analysed for VEGF.

### VEGF assay and Western blot analysis of platelet concentrates

Platelets were prepared from a 7-day-old platelet concentrate (prepared from platelet-rich plasma) obtained from the Blood Transfusion Service. The platelet suspension (155 ml, $1.542 \times 10^{12}$ plts $l^{-1}$, $0.28 \times 10^{9}$ WBC $l^{-1}$) was centrifuged at 2000 g for 10 min at 20°C, the supernatant removed and assayed for VEGF and the platelet pellet washed 2× with Tris buffered saline (50 mM Tris-HCl, 150 mM sodium chloride, pH 7.4; TBS) containing 5 mM glucose and 0.129 M sodium citrate. The platelet pellet was then resuspended in 1 ml of TBS containing 5 mM glucose and an excess of thrombin (320 U) added to ensure maximum release. After activation, the releasate was assayed for VEGF. For Western blotting analysis, supernatant from stored platelets diluted with an equal volume of phosphate-buffered saline (PBS) and containing approximately 100 ng of VEGF as measured by immunoassay was incubated for 1 h at 4°C with 1 ml of heparin sepharose (Pharmacia). After incubation, the heparin sepharose was collected by centrifugation, washed once with PBS and twice with PBS containing 0.5 M sodium chloride, resuspended in 200 ul of Laemmli electrophoresis sample buffer containing 2% (w/v) sodium dodecyl sulphate (SDS) and 5% v/v 2-mercaptoethanol, boiled for 4 min and 75 ul per lane was electrophoresed on a 12% SDS–polyacrylamide gel (SDS-PAGE). After electrophoresis, the gel was electroblotted onto nitrocellulose membranes (Hybond C Super; Amersham, Little Chalfont, UK). Blots were blocked with TBS/0.1% (v/v) Tween 20 (TBS-T) containing 10% (w/v) dried skimmed milk, and VEGF was detected by sequential incubation with a polyclonal rabbit antibody to VEGF, raised against the first 20 N-terminal amino acids of VEGF (Santa Cruz Biotechnology; purchased from Autogen Bioclear, Devizes, UK) at 1 µg $ml^{-1}$, biotinylated sheep anti-rabbit immunoglobulins (Dako, UK) at 1:1000 dilution and peroxidase-conjugated streptavidin (Dako) at 1:3000. Blots were developed using enhanced chemiluminescence (ECL) reagents (Amersham, UK) with subsequent detection of light emitted using XAR5 film (Kodak). Washing between steps was carried out using TBS-T and all antibodies were diluted in TBS-T containing 1% (w/v) dried skimmed milk. Fifty ng of recombinant human VEGF (Peprotech, London, UK) was electrophoresed as a positive control and molecular sizes of protein bands were determined by parallel electrophoresis of biotinylated molecular weight markers (Biorad, Hemel Hempstead, UK). Non-specific binding of antibodies was determined by parallel incubation of an identical blot with an irrelevant primary antibody in place of the rabbit anti-VEGF. The anti-VEGF antibody specifically reacts with VEGF and does not detect related molecules, such as PDGF, placental growth factor, VEGF-B or VEGF-C (information supplied by the manufacturer).

**Figure 1** Circulating platelet number vs serum VEGF concentration in eight normal donors. Clotted non-coagulated blood (●) and clotted citrated blood (○)

### Immunocytochemistry

Platelet cytospins and normal bone marrow smears were air dried and fixed in acetone for 5 min. Paraffin-embedded sections of a trephine biopsy from a patient with idiopathic thrombocytopenia purpura were dewaxed in xylene and subsequently rehydrated by passage through alcohols to water. Specimens were then incubated with 20% (v/v) normal goat serum in TBS for 5 min before addition of rabbit anti-VEGF (raised against the first 20 N-terminal amino acids; Santa Cruz Biotechnology) at 2.5 µg $ml^{-1}$ in TBS/0.1% (w/v) human serum albumin/0.1% (w/v) sodium azide. Endogenous avidin–biotin binding sites were blocked using the avidin–biotin blocking kit (Vector). After 1 h, specimens were washed in TBS and incubated in biotinylated goat anti-rabbit immunoglobulins (Vector) at 2.5 µg $ml^{-1}$ for 30 min. Labelling was visualized using a Vectastain avidin–biotin alkaline phosphatase detection system with Vector Red substrate (Vector) according to standard protocols. Specimens were stained with haematoxylin for 1 min and mounted or dehydrated and mounted as appropriate. Specificity controls included omission of the primary antibody, use of an irrelevant primary antibody and prior overnight adsorption of the primary antibody with control VEGF peptide at 25 µg $ml^{-1}$ (Santa Cruz Biotechnology).

### Determination of optimal sample processing for assay of circulating VEGF

In order to study the effect of sample-handling time and anticoagulant on VEGF levels in blood, 35 ml of venous blood was taken from each of four volunteers and divided into 16 1.3-ml plastic microtubes (Sarstedt) containing either EDTA (potassium salt), lithium heparin, trisodium citrate or no anticoagulant (four of each). Blood was also added to one each of a 3-ml plastic EDTA tube, a 3-ml plastic citrate tube, a 3-ml glass citrate tube and a 4-ml glass EDTA tube. Serum and plasma were then prepared from one of each type of tube by centrifuging at 2000 *g* for 10 min, immediately and at 30 min, 1 h (larger plastic and glass tubes), 2 h and 4 h after blood sampling. Serum and plasma samples were aliquoted and stored at –70°C until analysis. In light of the results, further blood samples were taken from four additional volunteers into glass citrate and EDTA tubes and plastic EDTA tubes and plasma prepared at 30 min and 1 h after collection. In addition, plasma was prepared from blood samples 1 h after collection (matched glass citrate and EDTA tubes) from five patients with breast cancer and stored at –70°C until analysed.

© Cancer Research Campaign 1998

**Figure 2** Theoretical VEGF production by platelets (serum and platelet-rich plasma samples from eight normal donors)

**Figure 3** Western blotting of platelet supernatant (**a** and **b**) and recombinant VEGF (**c** and **d**) by 12% SDS-PAGE under reducing conditions using rabbit anti-VEGF as the primary antibody (**a** and **c**). the corresponding negative controls (**b** and **d**) using an irrelevant rabbit primary antibody are shown

**Figure 4** Immunostaining (red colour) of a bone marrow biopsy (**A** and **B**; × 320) and a megakaryocyte from a bone marrow smear (**C**; × 1000) using a rabbit anti-VEGF antibody (**A** and **C**) or irrelevant rabbit immunoglobulin (**B**)

### Statistical analyses

Comparison of the effects of different types of sample-handling on VEGF concentration was carried out using one-way analysis of variance (ANOVA) followed by modified *t*-test with Bonferroni correction on those groups showing significant changes by ANOVA, using the statistical package SPSS-PC.

### RESULTS

### Assessment of VEGF recovery in serum and plasma samples and parallelity with the standard curve

Recoveries of VEGF in the serum and plasma samples tested were similar ranging from 94.1% to 112.6% with the lower spike and from 106.1% to 119.7% with the higher spike. Similar recoveries were also obtained with citrated plasma samples. Serial dilutions of the spiked plasma and serum samples diluted out in parallel to the standard curve (data not shown). Coefficients of variation of samples assayed in duplicate were generally less than 5%.

### VEGF release in blood samples

The VEGF concentrations of the blood samples from the eight volunteers after the various treatments are shown in Table 2. Values have been corrected for the dilution effect of the citrate anticoagulant and thrombin addition when appropriate, taking into account the haematocrit, i.e. assuming the citrate partitions into the liquid phase. Haematological analysis of the platelet-rich preparations showed them to be 99–100% pure. Serum VEGF concentrations ranged from 76 to 854 pg $ml^{-1}$ and were significantly higher ($P < 0.01$) than the matched citrated plasma VEGF concentrations, which ranged from < 9 to 42 pg $ml^{-1}$. Clotting of the platelet-free plasma produced little change (range 18–43 pg $ml^{-1}$). However, clotting of PRP with thrombin resulted in higher VEGF levels of 38–505 pg $ml^{-1}$, which were significantly correlated with serum values ($r_s = 0.88$, $P = 0.004$), and clotting of citrated whole blood by recalcification resulted in VEGF concentrations of 56–372 pg $ml^{-1}$, which were also significantly correlated with serum values ($r_s = 0.98$, $P < 0.001$). Neither serum VEGF nor clotted citrate blood VEGF levels were significantly correlated with any haematological parameter examined, just failing however to achieve a significant correlation with

© Cancer Research Campaign 1998

**Figure 5** VEGF concentrations of plasma and serum samples from four healthy volunteers (**A–D**). The different types of anticoagulant are as indicated with the time between venepuncture and sample processing indicated in hours (0–4). Bars show the standard deviations and asterisks indicate the results that are statistically significantly different from the zero time point for each anticoagulant (*$P < 0.05$, **$P < 0.01$, ***$P < 0.001$)

platelet number [$r_s = 0.62$ ($P = 0.1$) and 0.71 ($P = 0.07$) respectively; Figure 1]. As a test of the hypothesis that platelets are a major source of the elevated VEGF in serum samples, the VEGF concentrations found after activation of PRP were calculated in terms of the number of platelets present, and a similar calculation was performed for the directly prepared serum samples. A significant correlation ($r_s = 0.95$, $P < 0.001$) was found between the theoretical platelet-derived VEGF in serum and PRP after platelet activation (Figure 2) with mean (s.d.) values of 0.64 (0.41) and 0.56 (0.36) pg per $10^6$ platelets respectively.

### VEGF assay and Western blot analysis of platelet concentrates

In the releasate of the thrombin-activated platelet concentrate (containing $240 \times 10^9$ platelets), a total of 27 ng of VEGF was detected by immunoassay, which was approximately 20% of that expected on the basis of the above theoretical platelet-derived VEGF concentrations found with PRP and serum. Assay of the cell-free supernatant (155 ml) from the apheresis bag showed VEGF to be present at a concentration of 892 pg $ml^{-1}$, representing a further 138 ng of VEGF in total. Assuming this to be platelet-derived, the total VEGF content of 165 ng represents a theoretical platelet VEGF content of 0.69 pg per $10^6$ platelets, a value similar to those found using serum and PRP. Using Western blotting, a single band of approximately 25 kDa was seen in the platelet supernatant (Figure 3) compared with a band of approximately 22 kDa for the non-glycosylated recombinant VEGF. No bands were seen in the non-specific controls.

### Immunocytochemistry

No positive staining for VEGF was observed for the platelet cytospins. However, using the bone marrow samples, megakaryocytes stained positively for VEGF with a granular cytoplasmic appearance. Heterogeneous staining of some other cells, including some myeloid cells and stromal elements, was seen but cells of the erythroid lineage generally appeared to be negative (Figure 4). No positive labelling of cells was seen with omission of the primary antibody, replacement of the primary antibody with an irrelevant rabbit antibody or after prior incubation of the anti-VEGF antibody with control VEGF peptide.

© Cancer Research Campaign 1998

**Figure 6** A comparison of VEGF concentrations of plasma prepared with EDTA and citrate as anticoagulant in either plastic or glass tubes processed at 0.5 and 1 h post venepuncture. P, plastic, G, glass

### Determination of optimal sample processing for immunoassay of circulating VEGF

The effects of different anticoagulants and time before sample processing for four healthy volunteers are shown in Figure 5. Interindividual differences exist with significant increases in VEGF concentration occurring with increasing time before sample handling for clotted samples, and EDTA anticoagulated samples in two people. No significant change with time was seen in heparinized samples and a significant increase was seen in citrated plasma levels in only one individual and then only after 2 h. Using the 1 h time point as a comparison for each individual and examining the effects of anticoagulants relative to the citrate, which should be expected to have minimal effect on platelet activation, three of the four volunteers had significantly higher serum VEGF concentrations (Figure 5B, $P < 0.01$; Figure 5C, $P < 0.0001$; Figure 5D, $P < 0.001$). EDTA and heparinized plasma VEGF concentrations were significantly higher in one volunteer (Figure 5C, $P < 0.0001$ and $P < 0.001$ respectively) although EDTA plasma VEGF concentrations in one further volunteer (Figure 5A) were higher than those of the citrate sample but just failed to reach statistical significance because of the relatively high standard deviation of the citrate VEGF sample at this time point in this volunteer (although being significantly higher at 30 min and 1 h).

The initial comparison of glass and plastic citrate and EDTA tubes at 1 h showed no significant difference between glass and plastic tubes for VEGF concentrations in citrated plasma. However, the use of glass EDTA tubes rather than plastic appeared to minimize any increase in VEGF in three of the individuals. When this was investigated further in four more individuals, with VEGF concentrations in EDTA plasma from plastic and glass tubes being compared with VEGF concentrations in citrated plasma using glass tubes (samples processed at 30 min and 1 h post-venepuncture), no difference was seen with regard to time (Figure 6). However, using the 1-hour time point for comparison purposes, the use of plastic EDTA tubes resulted in significantly higher VEGF concentrations in all four individuals ($P < 0.05$ or $P < 0.01$), whereas the use of glass EDTA tubes produced a result significantly higher than that of citrated plasma in only two of the four individuals.

When the VEGF concentrations of the matched citrate and EDTA plasma samples (glass tubes) from the eight healthy volunteers were examined, together with those from five patients with breast cancer, VEGF concentrations of < 9–61 pg ml$^{-1}$ and < 9–149 pg ml$^{-1}$, respectively, were found (mean ± s.d. of 27.0 ± 14.0 and 55.2 ± 39.6 respectively). The differences between the two types of sample was statistically significant ($P = 0.004$), although the magnitude of the difference varied considerably between individuals (from 0 to 88 pg ml$^{-1}$).

### DISCUSSSION

Angiogenesis is important in both physiological and pathological processes and the pivotal role of VEGF in angiogenesis and vasculogenesis is clearly illustrated by the abnormality of blood vessel development and embryonic lethality resulting from the loss of even a single VEGF allele (Carmeliet et al, 1996; Ferrara et al, 1996). The regulation of the release of such a cytokine is obviously critical, and the finding here that platelets carry a readily available store of VEGF has implications for early events in several processes involving platelet–endothelial cell interactions, such as wound healing, atherosclerosis and tumour metastasis. Platelets have been implicated in tumour metastasis after observations of circulating tumour cells forming aggregates with platelets, decreased metastatic spread of tumour cells in thrombocytopenic mice compared with normal animals or after treatments that decrease platelet–tumour cell interactions and additionally the procoagulant activity of many tumours leading to activation of platelets (Blood and Zetter, 1990). Clearly the relevance of platelet-derived VEGF is dependent on its local release and the number of platelets involved and the overall balance with other platelet-derived angiogenic or anti-angiogenic factors, such as platelet-derived endothelial cell growth factor (thymidine phosphorylase), transforming growth factor-β (TGF-β), interleukin 1 (IL-1) and platelet activating factor-4 (Harrison and Cramer, 1993).

The results here clearly show that the elevated levels of VEGF in serum are as a result of its release from platelets during the clotting process. Although initially conceivable that matrix effects of serum and plasma or release of protein-sequestered VEGF by protease release during coagulation could account for such effects, the significant correlations between VEGF concentrations of serum and clotted platelet-rich plasma but not platelet-free plasma and those between the theoretical platelet-derived VEGF of serum and platelet-rich plasma would support a platelet-derived source of VEGF. Although platelet number just failed to reach a statistically significant correlation with serum VEGF concentration, this is probably explained by the interindividual variation in platelet VEGF content as exemplified in Figure 2. Such a wide interindividual variability in cellular production of cytokines is not unexpected, also being the case, for example, for platelet content of TGF-β (Jiang et al, 1995). Using immunoassay and Western blotting, the presence of VEGF in platelets was also confirmed by the detection of VEGF in supernatant from purified platelets after activation or prolonged storage, and its presence in megakaryocytes demonstrated immunocytochemically. These results have implications for sample-handling if accurate VEGF measurements are to be made. The material of choice would be citrated plasma processed within 1 h of venepuncture, although heparinized samples may represent a possible alternative and, although not explored here, the addition of inhibitors of platelet degranulation to the blood collection tubes may also be a consideration. Although similar results were obtained for EDTA plasma and citrated plasma in some cases, marked interindividual differences exist, with significant elevations being seen in some individuals, presumably because of differing degrees of platelet activation by EDTA, which were less marked if glass tubes were used. The reason for higher levels of VEGF in one EDTA sample compared with the corresponding serum sample (Figure 5), which would be assumed to contain the maximum level of platelet-derived VEGF after platelet activation, is unclear but may reflect release from other cells (e.g. monocytes) or release from a calcium-dependent sequestration, for example a binding protein. Clearly serum is totally unsuitable, with results having the potential to be markedly influenced by platelet number and VEGF content. To date, only a small number of studies have been carried out measuring circulating VEGF concentrations and all have used serum samples (Kondo et al, 1994; Baker et al, 1995; Hanatani et al, 1995; Ferrari and Scagliotti, 1996; Takano et al, 1996; Watanabe et al, 1996; Dirix et al, 1997; Lyall et al, 1997). Additional factors to be considered when measuring VEGF in clinical samples should include the specificity of the antibodies used in the assay and the possible presence and effect of binding of VEGF to heparin-like molecules, soluble receptors (Kendall and Thomas, 1993; Boocock et al, 1995) and $\alpha_2$-macroglobulin (Soker et al, 1993).

The question of release of VEGF from platelet concentrates during storage and its biological significance should be addressed. Clearly, in the platelet concentrate sample examined here, significant amounts of VEGF were present in the fluid phase after 7 days storage. However, whether such release of VEGF occurs within the normal shelf-life of such concentrates and whether it is biologically active remains to be determined. Contaminating cytokines, such as TNF-α, IL-1, IL-8 and IL-6, have been described in platelet concentrates prepared from platelet-rich plasma and implicated in transfusion reactions (Muylle et al, 1993; Stack and Snyder, 1994) although thought to be derived from contaminating white blood cells, such as monocytes, and significantly reduced when prefiltered (Muylle and Peetermans, 1994; Aye et al, 1995). A reduction in adverse reactions was also seen when platelet concentrates derived from buffy coats were used (Oksanen et al, 1994), and studies have reported that white cell contamination in such preparations is low and cytokine contamination is rare (Flegel et al, 1995; Kluter et al, 1995).

Using immunohistochemical techniques, VEGF appears to be present in megakaryocytes, with labelling being granular in appearance. The inability to detect VEGF in platelets by light microscopy was to be expected, given that the theoretical platelet-derived VEGF amounts to approximately five molecules per cell. The immunocytochemical demonstration of VEGF in megakaryocytes would not in itself be conclusive proof of synthesis by these cells as endocytic pathways for proteins, such as albumin and IgG, have been demonstrated (Harrison and Cramer, 1993). However, while this study was in progress, a recent report has described the presence of mRNA for VEGF in platelets and megakaryocytes (Katoh et al, 1995). Ultrastructural and release studies are needed to demonstrate whether the VEGF released by platelets is contained within cytoplasmic granules or is localized to the membranes, as is the case for IL-1 (Hawrylowicz et al, 1989).

The presence of mRNA for VEGF has also been described in T lymphocytes (Freeman et al, 1995), CD34$^+$ cells and monocytes (Katoh et al, 1995), with mRNA for the KDR and Flt-1 genes encoding VEGF receptors also present in CD34$^+$ cells, megakaryocytes and platelets (Katoh et al, 1995). These results support the immunohistochemical demonstration here of VEGF protein within several cell types of the bone marrow. The role of VEGF within the bone marrow is not clear, but the simultaneous presence of VEGF and its receptors on several haematopoietic cell types may indicate an autocrine or paracrine function in regulating growth or differentiation of these cells, in addition to the regulation of marrow endothelial cells. In further support of this, VEGF was

© Cancer Research Campaign 1998

found to enhance colony formation by mature subsets of granulocyte–macrophage and erythroid progenitor cells stimulated with a colony-stimulating factor but inhibited colony formation by more immature subsets of progenitors. In single-cell assays, these effects were absent or reduced implying both direct and indirect actions of VEGF (Broxmeyer et al, 1995). VEGF also markedly suppressed apoptotic cell death of normal human haematopoietic stem cells or leukaemic cell lines after gamma-ray irradiation (Katoh et al, 1995), supporting a potential role in survival and maintenance of these cells.

Our observations suggest additional possible mechanisms for the role of VEGF in cancer biology and prognosis. The adherence of circulating tumour cells to platelets may result in platelet activation and release of VEGF. This may result in increased endothelial permeability, allowing cellular extravasation, and local release of VEGF may stimulate neovascularization.

### ACKNOWLEDGEMENTS

The support of the Imperial Cancer Research Fund is gratefully acknowledged. We are grateful to Lynne Hill for technical assistance with the immunohistochemistry.

### NOTE ADDED IN PROOF

The production and release of VEGF by platelets has been demonstrated by Mohle et al since submission of this article. Mohle et al (1997) *Proc Natl Acad Sci USA* **94**: 663–668.

### REFERENCES

Abu-Jawdeh GM, Faix JD, Niloff J, Tognazzi K, Manseau E, Dvorak HF and Brown LF (1996) Strong expression of vascular permeability factor (vascular endothelial growth factor) and its receptors in ovarian borderline and malignant neoplasms. *Lab Invest* **74**: 1105–1115

Anan K, Morisaki T, Katano M, Ikubo A, Kitsuki H, Uchiyama A, Kuroki S, Tanaka M and Torisu M (1996) Vascular endothelial growth factor and platelet-derived growth factor are potential angiogenic and metastatic factors in human breast cancer. *Surgery* **119**: 333–339

Asano M, Yukita A, Matsumoto T, Kondo S and Suzuki H (1995) Inhibition of tumor growth and metastasis by an immunoneutralizing monoclonal antibody to human vascular endothelial growth factor vascular permeability factor$_{121}$. *Cancer Res* **55**: 5296–5301

Aye MT, Palmer DS, Giulivi A and Hashemi S (1995) Effect of filtration of platelet concentrates on the accumulation of cytokines and platelet release factors during storage. *Transfusion* **35**: 117–124

Baker PN, Krasnow J, Roberts JM and Yeo KT (1995) Elevated serum levels of vascular endothelial growth factor in patients with preeclampsia. *Obstet Gynecol* **86**: 815–821

Banks RE, Forbes MA, Stanley A, Kinsey S, Ingham E, Walters C and Selby PJ (1996) Presence of vascular endothelial growth factor (VEGF) in megakaryocytes and platelets (abstract). *Br J Haematol* **93**: A78

Barleon B, Sozzani S, Zhou D, Weich HA, Mantovani A and Marmé D (1996) Migration of human monocytes in response to vascular endothelial growth factor (VEGF) is mediated via the VEGF receptor flt-1. *Blood* **87**: 3336–3343

Blood CH and Zetter BR (1990) Tumor interactions with the vasculature: angiogenesis and tumor metastasis. *Biochim Biophys Acta* **1032**: 89–118

Boocock CA, Charnock-Jones DS, Sharkey AM, McLaren J, Barker PJ, Wright KA, Twentyman PR and Smith SK (1995) Expression of vascular endothelial growth factor and its receptors flt and KDR in ovarian carcinoma. *J Natl Cancer Inst* **87**: 506–516

Brown LF, Berse B, Jackman RW, Tognazzi K, Manseau EJ, Dvorak HF and Senger DR (1993*a*) Increased expression of vascular permeability factor (vascular endothelial growth factor) and its receptors in kidney and bladder carcinomas. *Am J Pathol* **143**: 1255–1262

Brown LF, Berse B, Jackman RW, Tognazzi K, Manseau EJ, Senger DR and Dvorak HF (1993*b*) Expression of vascular permeability factor (vascular endothelial growth factor) and its receptors in adenocarcinomas of the gastrointestinal tract. *Cancer Res* **53**: 4727–4735

Brown LF, Berse B, Jackman RW, Tognazzi K, Guidi AJ, Dvorak HF, Senger DR, Connolly JL and Schnitt SJ (1995) Expression of vascular permeability factor (vascular endothelial growth factor) and its receptors in breast cancer. *Hum Pathol* **26**: 86–91

Broxmeyer HE, Cooper S, Li ZH, Lu L, Song HY, Kwon BS, Warren RE and Donner DB (1995) Myeloid progenitor cell regulatory effects of vascular endothelial cell growth factor. *Int J Haematol* **62**: 203–215

Carmeliet P, Ferreira V, Breier G, Pollefeyt S, Lieckens L, Gertsenstein M, Fahrig M, Vandenhoeck A, Harpal K, Eberhardt C, Declerq C, Pawling J, Moons L, Collen D, Risau W and Nagy A (1996) Abnormal blood vessel development and lethality in embryos lacking a single VEGF allele. *Nature* **380**: 435–439

Claffey KP, Brown LF, Del Aguila LF, Tognazzi K, Yeo KT, Manseau EJ and Dvorak HF (1996) Expression of vascular permeability factor vascular endothelial growth factor by melanoma cells increases tumor growth, angiogenesis, and experimental metastasis. *Cancer Res* **56**: 172–181

De Vries C, Escobedo JA, Ueno H, Houck K, Ferrara N and Williams LT (1992) The fms-like tyrosine kinase, a receptor for vascular endothelial growth factor. *Science* **255**: 989–991

Diaz-Flores L, Gutierrez R and Varela H (1994) Angiogenesis: an update. *Histol Histopathol* **9**: 807–843

Dirix LY, Vermeulen PB, Pawinski A, Prove A, Benoy I, De Pooter C, Martin M and Van Oosterom (1997). Elevated levels of the angiogenic cytokines basic fibroblast growth factor and vascular endothelial growth factor in sera of cancer patients. *Br J Cancer* **76**: 238–243

Dvorak HF, Brown lF, Detmar M and Dvorak AM (1995) Vascular permeability factor/vascular endothelial growth factor, microvascular hyperpermeability, and angiogenesis. *Am J Pathol* **146**: 1029–1039

Ferrara N, Heinsohn H, Walder CE, Bunting S and Thomas GR (1995) The regulation of blood vessel growth by vascular endothelial growth factor. *Ann NY Acad Sci* **752**: 246–256

Ferrara N, Carver-Moore K, Chen H, Dowd M, Lu L, O'Shea KS, Powell-Braxton L, Hillan KJ and Moore MW (1996) Heterozygous embryonic lethality induced by targeted inactivation of the VEGF gene. *Nature* **380**: 439–442

Ferrari G and Scagliotti GV (1996) Serum and urinary vascular endothelial growth factor levels in non-small cell lung cancer patients. *Eur. J. Cancer* **32A**: 2368–2369

Flegel WA, Wiesneth M, Stampe D and Koerner K (1995) Low cytokine contamination in buffy coat-derived platelet concentrates without filtration. *Transfusion* **35**: 917–920

Folkman J (1995) Clinical applications of research on angiogenesis. *New Engl J Med* **333**: 1757–1763

Folkman J and Shing Y (1992) Angiogenesis. *J Biol Chem* **267**: 10931–10934

Freeman MR, Schneck FX, Gagnon ML, Corless C, Soker S, Niknejad K, Peoples GE and Klagsbrun M (1995) Peripheral blood T lymphocytes and lymphocytes infiltrating human cancers express vascular endothelial growth factor: A potential role for T cells in angiogenesis. *Cancer Res* **55**: 4140–4145

Grimmond S, Lagercrantz J, Drinkwater C, Silins G, Townson S, Pollock P, Gotley D, Carson E, Rakar S, Nordenskjöld M, Ward L, Hayward N and Weber G (1996) Cloning and characterization of a novel human gene related to vascular endothelial growth factor. *Genome Res* **6**: 124–131

Guidi AJ, Fischer L, Harris JR and Schnitt SJ (1994) Microvessel density and distribution in ductal carcinoma in situ of the breast. *J Natl Cancer Inst* **86**: 614–619

Hanatani M, Tanaka Y, Kondo S, Ohmori I and Suzuki H (1995) Sensitive chemiluminescence enzyme immunoassay for vascular endothelial growth factor vascular permeability factor in human serum. *Biosci Biotechnol Biochem* **59**: 1958–1959

Harrison P and Cramer EM (1993) Platelet alpha-granules. *Blood Rev* **7**: 52–62

Hauser S and Weich HA (1993) A heparin-binding form of placenta growth factor (PlGF-2) is expressed in human umbilical vein endothelial cells and in placenta. *Growth Factors* **9**: 259–268

Hawrylowicz CM, Santoro SA, Platt FM and Unanue ER (1989) Activated platelets express IL-1 activity. *J Immunol* **143**: 4015–4018

Houck KA, Ferrara N, Winer J, Cachianes G, Li B and Leung DW (1991) The vascular endothelial growth factor family: identification of a fourth molecular species and characterization of alternative splicing of RNA. *Mol Endocrinol* **5**: 1806–1814

Houck KA, Leung DW, Rowland AM, Winer J and Ferrara N (1992) Dual regulation of vascular endothelial growth factor bioavailability by genetic and proteolytic mechanisms. *J Biol Chem* **267**: 26031–26037

© Cancer Research Campaign 1998 

Jiang X, Kanai H, Hiromura K, Sawamura M and Yano S (1995) Increased intraplatelet and urinary transforing growth factor-β in patients with multiple myeloma. *Acta Haematol* **94**: 1–6

Joukov V, Pajusola K, Kaipainen A, Chilov D, Lahtinen I, Kukk E, Saksela O, Kalkkinen N and Alitalo K (1996) A novel vascular endothelial growth factor, VEGF-C, is a ligand for the Flt4 (VEGFR-3) and KDR (VEGFR-2) receptor tyrosine kinases. *Embo J* **15**: 290–298

Katoh O, Tauchi H, Kawaishi K, Kimura A and Satow Y (1995) Expression of the vascular endothelial growth factor (VEGF) receptor gene, KDR, in hematopoietic cells and inhibitory effect of VEGF on apoptotic cell death caused by ionizing radiation. *Cancer Res* **55**: 5687–5692

Kendall RL and Thomas KA (1993) Inhibition of the vascular endothelial cell growth factor activity by an endogenously encoded soluble receptor. *Proc Natl Acad Sci USA* **90**: 10705–10709

Kluter H, Muller-Steinhardt M, Danzer S, Wilhelm D and Kirchner H (1995) Cytokines in platelet concentrates prepared from pooled buffy coats. *Vox Sang* **69**: 38–43

Kondo S, Asano M, Matsuo K, Ohmori I and Suzuki H (1994) Vascular endothelial growth factor/vascular permeability factor is detectable in the sera of tumor-bearing mice and cancer patients. *Biochim Biophys Acta* **1221**: 211–214

Lyall F, Greer IA, Boswell F and Fleming R (1997) Suppression of serum vascular endothelial growth factor immunoreactivity in normal pregnancy and in pre-eclampsia. *Br J Obstet Gynaecol* **104**: 223–228

Maeda K, Chung YS, Ogawa Y, Takatsuka S, Kang SM, Ogawa M, Sawada T and Sowa M (1996) Prognostic value of vascular endothelial growth factor expression in gastric carcinoma. *Cancer* **77**: 858–863

Maglione D, Guerriero V, Viglietto G, Delli-Bovi P and Persico MG (1991) Isolation of a human placenta cDNA coding for a protein related to the vascular permeability factor. *Proc Natl Acad Sci USA* **88**: 9267–9271

Maglione D, Guerriero V, Viglietto G, Ferraro MG, Aprelikova O, Alitalo K, Del Vecchio S, Lei KJ, Chou JY and Persico MG (1993) Two alternative mRNAs coding for the angiogenic factor, placenta growth factor (PlGF), are transcribed from a single gene of chromosome 14. *Oncogene* **8**: 925–931

Mattern J, Koomägi R and Volm M (1996) Association of vascular endothelial growth factor expression with intratumoral microvessel density and tumour cell proliferation in human epidermoid lung carcinoma. *Br J Cancer* **73**: 931–934

Matthews W, Jordan CT, Gavin M, Jenkins NA, Copeland NG and Lemischka IR (1991) A receptor tyrosine kinase cDNA isolated from a population of enriched primitive hematopoietic cells and exhibiting close genetic linkage to c-kit. *Proc Natl Acad Sci USA* **88**: 9026–9030

Millauer B, Wizigmann-Voos S, Schnurch H, Martinez R, Moller NP, Risau W and Ullrich A (1993) High affinity VEGF binding and developmental expression suggest Flk-1 as a major regulator of vasculogenesis and angiogenesis. *Cell* **72**: 835–846

Muylle L and Peetermans ME (1994) Effect of prestorage leukocyte removal on the cytokine levels in stored platelet concentrates. *Vox Sang* **66**: 14–17

Muylle L, Joos M, Wouters E, De Bock R and Peetermans ME (1993) Increased tumor necrosis factor α (TNFα), interleukin 1, and interleukin 6 (IL-6) levels in the plasma of stored platelet concentrates: relationship between TNFα and IL-6 levels and febrile transfusion reactions. *Transfusion* **33**: 195–199

O'Brien T, Cranston D, Fuggle S, Bicknell R and Harris AL (1995) Different angiogenic pathways characterize superficial and invasive bladder cancer. *Cancer Res* **55**: 510–513

Oksanen K, Ebeling F, Kekomaki R, Elonen E, Sahlstedt L, Volin L and Myllyla G (1994) Adverse reactions to platelet transfusions are reduced by use of platelet concentrates derived from buffy coat. *Vox Sang* **67**: 356–361

Olofsson B, Pajusola K, Kaipainen A, Von Euler G, Joukov V, Saksela O, Orpana A, Petersson RF, Alitalo K and Eriksson U (1996) Vascular endothelial growth factor B, a novel growth factor for endothelial cells. *Proc Natl Acad Sci USA* **93**: 2576–2581

Paavonen K, Horelli-Kuitunen N, Chilov D, Kukk E, Pennanen S, Kallioniemi OP, Pajusola K, Olofsson B, Eriksson U, Joukov V, Palotie A and Alitalo K (1996) Novel human vascular endothelial growth factor genes VEGF-B and VEGF-C localize to chromosomes 11q13 and 4q34, respectively. *Circulation* **93**: 1079–1082

Saleh M, Stacker SA and Wilks AF (1996) Inhibition of growth of C6 glioma cells *in vivo* by expression of antisense vascular endothelial growth factor sequence. *Cancer Res* **56**: 393–401

Sato K, Terada K, Takahashi S, Saito M, Moriyama M, Kakinuma H, Suzuki Y, Kato M and Kato T (1994) Frequent overexpression of vascular endothelial growth factor gene in human renal cell carcinoma. *Tohoku J Ex Med* **173**: 355–360

Senger DR, Galli SJ, Dvorak AM, Perruzzi CA, Harvey VS and Dvorak HF (1983) Tumor cells secrete a vascular permeability factor that promotes accumulation of ascites fluid. *Science* **219**: 983–985

Senger DR, Perruzzi CA, Feder J and Dvorak HF (1986) A highly conserved vascular permeability factor secreted by a variety of human and rodent tumor cell lines. *Cancer Res* **46**: 5629–5632

Shibuya M, Yamaguchi S, Yamane A, Ikeda T, Tojo A, Matsushime H and Sato M (1990) Nucleotide sequence and expression of a novel human receptor-type tyrosine kinase gene (flt) closely related to the fms family. *Oncogene* **5**: 519–524

Soker S, Svahn CM and Neufeld G (1993) Vascular endothelial growth factor is inactivated by binding to α2-macroglobulin and the binding is inhibited by heparin. *J Biol Chem* **268**: 7685–7691

Stack G and Snyder EL (1994) Cytokine generation in stored platelet concentrates. *Transfusion* **34**: 20–25

Takahashi A, Sasaki H, Kim SJ, Tobisu K, Kakizoe T, Tsukamoto T, Kumamoto Y, Sugimura T and Terada M (1994) Markedly increased amounts of messenger RNAs for vascular endothelial growth factor and placenta growth factor in renal cell carcinoma associated with angiogenesis. *Cancer Res* **54**: 4233–4237

Takahashi Y, Kitadai Y, Bucana CD, Cleary KR and Ellis LM (1995) Expression of vascular endothelial growth factor and its receptor, KDR, correlates with vascularity, metastasis, and proliferation of human colon cancer. *Cancer Res* **55**: 3964–3968

Takano S, Yoshii Y, Kondo S, Suzuki H, Maruno T, Shirai S, and Nose T (1996) Concentration of vascular endothelial growth factor in the serum and tumor tissue of brain tumor patients. *Cancer Res* **56**: 2185–2190

Terman BI, Carrion ME, Kovacs E, Rasmussen BA, Eddy RL and Shows TB (1991) Identification of a new endothelial cell growth factor receptor tyrosine kinase. *Oncogene* **6**: 1677–1683

Thomas KA (1996) Vascular endothelial growth factor, a potent and selective angiogenic agent. *J Biol Chem* **271**: 603–606

Tischer E, Mitchell R, Hartman T, Silva M, Gospodarowicz D, Fiddes JC and Abraham JA (1991) The human gene for vascular endothelial growth factor. Multiple protein forms are encoded through alternative exon splicing. *J Biol Chem* **266**: 11947–11954

Toi M, Kashitani J and Tominaga T (1993) Tumor angiogenesis is an independent prognostic indicator in primary breast carcinoma. *Int J Cancer* **55**: 371–374

Toi M, Hoshina S, Takayanagi T and Tominaga T (1994) Association of vascular endothelial growth factor expression with tumor angiogenesis and with early relapse in primary breast cancer. *J J Cancer Res* **85**: 1045–1049

Warren RS, Yuan H, Matli MR, Gillett NA and Ferrara N (1995) Regulation by vascular endothelial growth factor of human colon cancer tumorigenesis in a mouse model of experimental liver metastasis. *J Clin Invest* **95**: 1789–1797

Watanabe O, Arimura K, Kitajima I, Osame M and Maruyama I (1996) Greatly raised vascular endothelial growth factor (VEGF) in POEMS syndrome. *Lancet* **347**: 702

© Cancer Research Campaign 1998